# Comparison of the Functional Outcomes of Arthroscopic Anterior Cruciate Ligament Reconstruction through the All-inside and Outside-in Techniques: A Double-Blinded Randomized Controlled Trial

**DOI:** 10.1055/s-0044-1786726

**Published:** 2024-06-22

**Authors:** Paulson Varghese, Saroj Kumar Patra, Gurudip Das, Bishnu P. Patro, Gunjar Jain, Haridas M. P.

**Affiliations:** 1 Departamento de Ortopedia, All India Institute of Medical Sciences (AIIMS), Bhubaneswar, Índia; 2 Departamento de Trauma e Emergência, All India Institute of Medical Sciences (AIIMS), Bhubaneswar, Índia; 3 Departamento de Farmacologia, All India Institute of Medical Sciences (AIIMS), Bhubaneswar, Índia

**Keywords:** anterior cruciate ligament, anterior cruciate ligament reconstruction, arthroscopy, postoperative complications, randomized controlled trials as topic, rupture

## Abstract

**Objective** To compare the functional outcomes of anterior cruciate ligament (ACL) reconstruction with hamstring autograft (HA) through the all-inside (AI) technique with adjustable-loop cortical Endobutton (Smith & Nephew, Watford, Hertfordshire, England) on the sides of the femur and tibia and through the outside-in (OI) technique using an interference screw on the tibial side and a cortical Endobutton on the femoral side.

**Materials and Methods** The present is a double-blinded randomized controlled trial (RCT) of 44 patients undergoing arthroscopic ACL reconstruction from February 2019 to February 2022 in a tertiary care hospital. As per computer-based randomization, the patients were distributed into two groups: the AI and OI groups. Both groups were evaluated for 12 months using the Visual Analog Scale (VAS), the Lysholm Knee Scoring Scale, and part I (pain score) and part II (function score) of the Knee Society Score (KSS).

**Results** On postoperative day 2, the VAS score was significantly higher in the OI group ( *p* = 0.0001), but insignificant ( *p* = 0.807) at 6 weeks. At 3, 6, and 12 months of follow-up, the score on the Lysholm Knee Scoring Scale was significantly higher ( *p* = 0.001) in the AI group. At 6 months, both parts of the KSS showed a significant difference, with the AI group presenting a better outcome ( *p* = 0.04). However, at 12 months, the AI group presented a better score on part I of the KSS, but no differences were observed regarding part II.

**Conclusion** In a follow-up of 12 months, the patients submitted to the AI technique presented better outcome scores and pain relief than those submitted to the OI technique.

## Introduction

Arthroscopic reconstruction of the anterior cruciate ligament (ACL) presents advantages such as a less invasive procedure and an earlier recovery. Many hospitals are performing arthroscopic ACL reconstruction as day-care surgery. The procedure can be performed through the all-inside (AI) technique, which only depends on the adjustable loop, or through the outside-In (OI) technique with a complete tibial tunnel using an interference screw and adjustable loop. 1

In the OI technique, the graft is fixed with an interference screw on the tibial side and Endobutton (Smith & Nephew, Watford, Hertfordshire, England) on the femoral side. Biomechanical studies 2 have shown that the interference screws present low fixation strength due to graft slippage. Other worries associated with interference screws are graft maceration and the small tendon-to-bone contact for the biological incorporation of the graft. 2

In the AI technique, two bone sockets are made on either side of the graft instead of the complete bone tunnels. The autograft is whip-stitched on both sides, and the sutures are attached to the cortical Endobutton on either side. 3 Recently, the use of adjustable-loop cortical Endobutton in both femoral and tibial sides came into practice, which presents the advantage of an implant-free tendon graft for bone fixation in both the femoral and tibial sides, which augments the tendon-to-bone biological integration. 4 The concern with the AI technique is graft elongation, which leads to an increased gap between the two cortical Endobuttons, which causes recurrence of the laxity and instability in the knee joint; moreover, it can lead to graft slippage. 5 In the literature, there are few studies comparing both methods in terms of functional outcomes; only two randomized controlled trials 6 7 and two prospective studies 8 9 have compared these two techniques. No studies have compared both methods with the use of an autograft. The present randomized control trial aimed to compare the functional outcomes of ACL reconstruction using the AI and OI techniques.

## Materials and Methods

### Study Design

The present prospective randomized double-blinded study with 2 parallel arms was conducted at a tertiary care hospital from February 2019 to February 2022, and it was approved by the institutional Ethics Committee (IEC/AIIMS BBSR/PG THESIS/2018-19/38).

#### Inclusion Criteria

➢ Patients with ACL tear without improvement after the conservative management.
➢ Patients with knee flexion greater than 90°.
➢ Cases of ACL tear associated with medial or lateral meniscus tear injuries.
➢ Patients aged between 20 and 60 years.
➢ Patients with ACL tears with a history of injury within one year.

#### Exclusion Criteria

➢ Patients with multiligament knee injury.
➢ Patients with associated bony injuries of the lower limb and injury to the spine.
➢ Patients with chondral lesions of grade II or higher as per the Outerbridge classification.
➢ Revision ACL reconstruction.
➢ Patients with local skin lesions over the surgical site.

### Randomization and Allocation Concealment

Patients with ACL tears who fulfilled the inclusion criteria were recruited after providing written informed consent. The patients were randomized into two groups using an online randomization software ( www.randomization.com ). Gender, age, injured side, presence of meniscal or chondral lesions, as well as the duration of the injury were recorded. Two surgeons (BPP and SKP) performed the surgery as per allocation. The investigator (GD) who evaluated and followed up the patients pre- and postoperatively in the outpatient clinic was also blinded about the procedure performed. Postoperatively, the patients were assessed according to the postoperative protocol. The randomization sequence was performed by another investigator (VP) who was not involved in the surgery, recruitment, or postoperative assessment of the patients.

The sample size was calculated based on the mean difference of 2 with an alpha error of 5% (95% confidence interval). Taking the power of 80%, we found that the sample size was of 19 patients in each group. Adding 15% of attrition to 19, which is 3, the sample size obtained was of 22 subjects in each group.

## Surgical Technique

In all cases, routine diagnostic arthroscopy was performed using standard portals. Both semitendinosus and gracilis autografts were harvested for the OI technique, and only the semitendinosus graft was harvested for the AI technique. Femoral bone tunnels were made similarly in both techniques using standard jigs. However, the tibial tunnel for the AI technique was performed using a FlipCutter (Arthrex, Inc., Naples, FL, United States) drill, and, for the OI technique, a standard jig. In the AI technique, the whip-stitched autograft is held by cortical Endobutton on both the femoral and tibial sides, whereas in the OI technique it is is held by cortical Endobutton on the femoral side and an interference screw on the tibial side. Any associated meniscal or chondral lesions were debrided or partially resected along with index reconstruction. Following surgery, knee lavage was performed, followed by wound closure. An extended knee brace was used in both groups. After the procedure, all patients were sent to rehabilitation as per the post-operative protocol.

## Rehabilitation

All patients underwent an accelerated rehabilitation protocol with closed-chain range of motion (ROM) exercises and full weight bearing with the long knee brace from postoperative day two, and open-chain ROM exercises and full weight bearing without the knee brace after two weeks, when they were instructed to perform home-based rehabilitation as per the protocol. The patients followed the rehabilitation protocol and usually returned to sports at six months.

### Outcome Measures

Both groups of patients were evaluated before surgery and on the second and fifth days, second and sixth weeks, and third, sixth, and twelfth month after surgery. The scores on the Visual Analog Scale (VAS), the Lysholm Knee Scoring Scale, and the Knee Society Score (KSS) were evaluated in the third, sixth, and twelfth postoperative months using appropriate proforma by a blinded investigator (GD). Pain was classified based on the VAS score into mild (< 3), moderate (3 to 7), and severe (> 7). Linear regression analysis was performed regarding the VAS score on the second day (dependent variable) and the technique (independent variable).

**Fig. 1:**
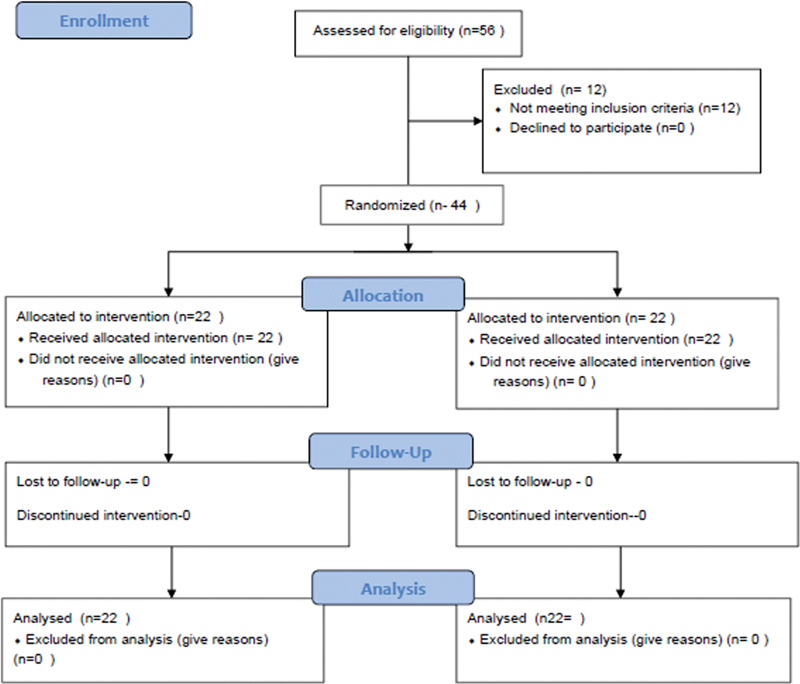
Consolidated Standards of Reporting Trials (CONSORT) diagram.

As per the power analysis, we included 44 (38 male and 6 female) patients randomly allocated into 2 groups (AI and OI) with 22 patients in each ( Fig. 1 ).

### Statistical Methodology

The statistical analysis was performed using the IBM SPSS Statistics for Windows (IBM Corp., Armonk, NY, United States) software.

## Results

### Demographics and Other Baseline Data

There was no significant difference regarding the demographic data (age, sex, and body mass index, BMI) or the other baseline data (duration of injury, laterality, physical activity, associated meniscal injury) between the two groups ( Table 1a ).

**Table 1a:** Baseline demographic data

	AI group (n = 22)	OI group (n = 22)
Male:female ratio	19:3	19:3
Age (years)	29	28.86
Duration of the injury (days)	150	159
Right:left ration	11:11	12:10
Mean body mass index (kg/m ^2^ )	24.79	24.79
Physical activity	Sedentary lifestyle: 2; average: 15; sportsperson5	Sedentary lifestuyle: 3; average: 15; sportsperson: 4
Associated meniscal injury	8	7

Abbreviations: AI, all-in technique; OI, outside-in technique.

The mean VAS score on the second postoperative day was significantly higher in the OI group ( *p* = 0.0001). However, the mean VAS scores of both groups in the sixth postoperative week were not significantly different ( *p* = 0.807) ( Table 1b ).

### Linear Regression Analysis

The linear regression analysis model was significant, with an R ^2^ value of 0.513 ( *p* < 0.05). The change in technique from AI to OI altered the VAS score by 2.995 ( Table 2 ). Sex, BMI, age, and physical activity were also compared regarding the scores on the VAS, Lysholm scale, and the KSS, but they did not result in a significant model.

**Table 1b:** Visual Analog Scale (VAS) scores at baseline, on day 2, and in 6 weeks of follow-up

Data	AI (mean ± SD)	OI (mean ± SD)	95%CI (lower-upper)		*p* -value
Baseline VAS	0.45 ± 0.510	0.41 ± 0.503	-0.263	-0.263	0.767
VAS day 2	1.32 ± 1.359	4.27 ± 1.579	-3.851	-2.058	0.0001
Mean difference	0.86 ± 1.521	3.86 ± 1.612	-3.963	-2.037	0.0001
VAS 6 weeks	0.14 ± 0.640	0.18 ± 0.588	-0.419	0.329	0.807
Mean difference	-0.32 ± 0.894	-0.23 ± 0.869	-0.627	0.445	0.734

Abbreviations: 95%CI, 95% confidence interval; AI, all-in technique; OI, outside-in technique; SD, standard deviation.

In the follow-up at the 3rd, 6th, and 12th postoperative months, the the score on the Lysholm scale ( *p* = 0.001) was significantly higher in the AI group ( Table 3 ). In this scale, the functional outcome is classified into poor (score < 65), fair (score ranging from 65 to 83), good (score ranging from 84 to 90) and excellent (score > 90). At three months, only 1 patient in the AI group had a poor score compared to five in the OI group, and 21 in the AI group had a fair score compared to 17 in the OI group. At 6 months, 1 patient in AI group had a fair score compared to 4 in the OI group; 9 patients had a good score in the AI group compared to 17 in the OI group; and 12 patients in the AI group had an excellent score compared to 1 in the OI group. At 12 months of follow-up, all patients except 1 in each group had superior functional outcome scores, and 1 in each group had a good score. The KSS has two parts: the pain score (part I) and the function score (part II). There was a significant difference in part I of the KSS at 3 months ( *p* = 0.009), but not in part II. At six months, both parts of the KSS showed a substantial difference between the two groups, with the AI group presenting a better score ( *p* = 0.04). However, at 12 months of follow-up, the AI group presented significantly better scores on part I of the KSS ( *p* = 0.033); regarding part II, there were no significant differences between the groups ( *p* = -0.543) ( Table 4 ). However, both groups improved significantly in the comparison between the preoperative assessments and the follow-up. The anterior drawer test and the Lachman test showed no significant differences in laxity between the two groups either preoperatively or at the follow-up. However, there was a substantial difference in laxity preoperatively and at follow-up in both groups according to the Lachman and anterior drawer tests.

**Table 2:** Linear regression analysis

	R ^2^	B	*p* -value
Technique	0.513	2.955	< 0.05
Visual Analog Scale score			

**Table 3:** Lysholm scale score at baseline and with 3, 6 and 12 months of follow-up

Data	AI (mean ± SD)	OI (mean ± SD)	*p* -value
Baseline	70.73 ± 0.456	70.64 ± 0.492	0.529
3 months	73.45 ± 4.906	68.14 ± 5.445	0.001
Mean difference after 3 months	2.73 ± 4.939	-2.50 ± 5.510	0.002
6 months	90.32 ± 4.202	85.64 ± 4.100	0.001
Mean difference after 6 months	19.59 ± 4.317	15.00 ± 4.220	0.001
12 months	97.95 ± 2.7	94.73 ± 3.33	0.001
Mean difference after 12 months	27.22 ± 0.116	24.09 ± 0.119	0.032

Abbreviations: AI, all-in technique; OI, outside-in technique; SD, standard deviation.

**Table 4:** Knee Society Score (KSS) at baseline and at 3, 6 and 12 months of follow-up

Data	AI (mean ± SD)	OI (mean ± SD)	*p* -value
KSS part I (baseline)	70.68 ± 0.477	70.59 ± 0.503	0.512
KSS part II (baseline)	69.68 ± 0.477	69.50 ± 0.512	0.230
KSS part I (3 months)	66.73 ± 5.978	60.73 ± 8.430	0.009
KSS part II (3 months)	51.64 ± 8.963	48.18 ± 9.825	0.230
Mean difference in KSS part I (3 months)	-3.95 ± 5.900	-9.86 ± 8.571	0.011
Mean difference in KSS part II (3 months)	-18.05 ± 8.941	-21.32 ± 9.544	0.247
KSS part I (6 months)	90.18 ± 2.986	86.73 ± 7.052	0.040
KSS part II (6 months)	88.23 ± 5.228	84.55 ± 6.710	0.049
Mean difference in KSS part I (6 months)	19.50 ± 2.988	16.14 ± 6.951	0.043
Mean difference in KSS part II (6 months)	18.55 ± 5.180	15.05 ± 6.586	0.050
KSS part I (12 months)	97.18 ± 3.14	94.14 ± 2.8	0.003
KSS part II (12 months)	98.45 ± 3.18	97.95 ± 2.5	0.556
Mean difference in KSS part I (12 months)	26.50 ± 1.9	23.55 ± 2.88	0.033
Mean difference in KSS part II (12 months)	28.81 ± 1.9	28.45 ± 2.11	0.543

**Abbreviations:** AI, all-in technique; OI, outside-in technique; SD, standard deviation.

### Complications

None of the patients underwent revision surgery. Six weeks postoperatively, 5 patients in the AI group (22.72%) and 12 in the OI group (54%) complained of altered sensation in the knee; 2 patients in the OI group developed foreign body sensation; and 1 patient in the AI group developed synovitis. Regarding joint effusion, 2 patients in the AI group and 3 in the OI group developed it. The foreign body sensation, synovitis, and joint effusion were resolved in every patient at 3 months of follow-up.

## Discussion

In the literature, there are few studies comparing the AI and OI techniques in terms of functional outcomes; only two randomized controlled trials 6 7 and two prospective studies 8 9 have compared them. Volpi et al. 9 reported that, concerning adequate articular function and return to sports, there are no differences between the AI technique and the traditional ACL reconstruction using the semitendinosus and gracilis tendons. Lubowitz et al. 6 reported no differences in the scores on the International Knee Documentation Committee (IKDC) Knee Examination Form, the KSS, and the 12-Item Short-Form Health Survey (SF-12), nor in terms of tibial and femoral widening, but that ACL reconstruction with the AI technique resulted in lower pain scores on the VAS compared with baseline. In another study, Lubowitz et al. 7 concluded that there were no significant differences in knee anteroposterior stability or other outcomes comparing AI ACL allograft reconstruction using aperture fixation and using suspensory fixation. The graft length requirement in the AI group ranged from 6 cm to 6.5 cm, and, in the OI group, it ranged from 8.5 cm to 9 cm. The requirement of graft length was lower in the AI group, so greater graft thickness is available for ligament reconstruction.

In a systematic review published in 2018, de Sa et al. 10 reported a better functional outcome and low graft failure rate with the AI technique. Browning et al. 11 reviewed ACL reconstruction using aperture fixation or suspensory fixation and found that suspensory fixation resulted in lower graft failure rates and better knee stability. However, in the present study, with the Lachman and anterior drawer tests, we observed no significant differences in knee stability between both groups, and none of our patients underwent revision surgery. The difference in functional outcome between the two methods was not significant in previous studies. 6 However, in the present study, with a medium follow-up of 12 months, better functional outcomes were observed in the AI group. We found that the AI technique results in significant pain relief from the immediate postoperative period until up to six weeks. After that, there were no significant differences in the VAS scores between the groups. Benea et al. 12 concluded that the pain level was lower in the AI group than in the classic cortical fixation group at one month follow-up. In 2015, Lubowitz et al. 7 compared suspensory fixation using femoral and tibial cortical buttons and aperture fixation using a femoral cannulated interference screw and a tibial cannulated interference retrograde screw; the follow up was of two years, and the “primary outcome measure was knee anteroposterior (AP) stability measured using the KT-1000 device (MEDmetric, San Diego, CA). Secondary outcome measures included change in pain score on a visual analog scale versus preoperatively, narcotic consumption, International Knee Documentation Committee knee examination rating, International Knee Documentation Committee subjective evaluation score, Knee Society Scores, Short Form 12 scores, and radiographic analysis for socket widening”. The authors 7 did not find any significant difference in any of these outcome scores. In 2014 Volpi et al. 9 compared pain and the functional outcome using the Tegner and Lysholm scales, and the IKDC score regarding reconstruction performed though the AI transtibial technique and the traditional transtibial technique with two years of follow-up, and they observed no significant changes in any of the outcomes. Return to sports is an important outcome measure after ACL reconstruction; however, it depends on the type of sport and rehabilitation protocol. The graft failure rates reported for AI ACL reconstruction in the literature 3 14 15 16 range from 4.9% to 12.7%; the mechanisms of failure in these studies were either trauma or sports-related injuries.

To conclude about the graft failure rate, the present study needs a longer follow-up. However, Connaughton et al. 3 reported similar overall results on subjective and objective outcome studies but a high rate of graft failure with the AI technique. However, their study 3 is biased because they took only allografts for the reconstruction, which have a high propensity to fail in young active individuals. Pallis et al. 17 observed high revision rates in ACL reconstructions using allografts and recommended autografts for ACL reconstruction in young athletes. In 2017, Schilaty et al. 18 assessed the incidence of second ACL injury and the risk factors associated with it, and found that allografts are associated with a higher risk of graft failure when compared with hamstring and bone-patellar tendon autografts. No conclusive data regarding graft failure rates comparing AI and OI techniques with autografts is available in the literature. However, there is an increased chance of graft failure using hamstring autografts if the graft diameter is shorter than 8 mm. 19 The OI technique uses semitendinosus and gracilis autografts, whereas the gracilis tendon is spared in the AI technique. Magnussen et al. 19 concluded that gracilis tendon harvest will negatively affect knee flexion isokinetic torque at a low angular velocity. This finding is significant for sports that require high functional activity; hence, the authors 19 recommended preservation of the gracilis tendon whenever possible. The AI technique also results in lower graft site morbidity since the gracilis is spared. 20 Kouloumentas et al. 21 reported better preservation of knee flexion strength in AI ACL reconstruction than with the conventional OI technique. Monaco et al. 22 stated that the technique that spares the gracilis is minimally-invasive for ACL reconstruction and yields better flexion strength at low angular velocity than the full tibial tunnel technique.

All patients of both groups had undergone accelerated rehabilitation. 23

The limitations of the present study are the relatively small sample size and the follow-up of only one year. This may explain the lack of graft failure among our patients.

Our findings show that the AI group presented lower levels of pain and better functional outcomes when followed for 12 months compared to the OI group, and that both techniques are successful in restorating knee ligamentous stability, and result in good patient-reported outcome measures and pain relief compared to the preoperative levels.

## Main Outcome

The AI group presented better functional outcomes compared to the OI group.

## Conclusion

In the present randomized controlled trial, we found that the AI and OI techniques with hamstring autograft for ACL reconstruction resulted in the restoration of knee stability and good patient-reported outcome measures. However, the AI group presented better scores on the Lysholm scale and pain relief in a follow-up of 12 months.
