# Supplementary material for: Comparison of the Functional Outcomes of Arthroscopic Anterior Cruciate Ligament Reconstruction through the All-inside and Outside-in Techniques: A Double-Blinded Randomized Controlled Trial
Source: Rev Bras Ortop (Sao Paulo). 2024 Jun 22;59(3):e385–92. doi: 10.1055/s-0044-1786726 (PMC11193577; doi:10.1055/s-0044-1786726)
Supplement: Related texts

# Research article: Comparação dos resultados funcionais da reconstrução artroscópica do ligamento cruzado anterior pelas técnicas *all-inside* e *outside-in* : Um ensaio clínico controlado, randomizado e duplo-cego

**Keywords:** artroscopia, complicações pós-operatórias, ensaios clínicos controlados e randomizados como assunto, ligamento cruzado anterior, reconstrução do ligamento cruzado anterior, ruptura

## Abstract

**Objetivo** Comparar os resultados funcionais da reconstrução do ligamento cruzado anterior (LCA) com autoenxerto de isquiotibiais pela técnica *all-inside* (AI) com Endobutton (Smith & Nephew, Watford, Hertfordshire, Inglaterra) cortical de alça ajustável nos lados do fêmur e da tíbia e pela técnica *outside-in* (OI) com parafuso de interferência no lado tibial e Endobutton cortical no lado femoral.

**Métodos** Trata-se de um ensaio clínico controlado, randomizado e duplo-cego com 44 pacientes submetidos à reconstrução artroscópica do LCA de fevereiro de 2019 a fevereiro de 2022 em um hospital de cuidados terciários. De acordo com a randomização por computador, os pacientes foram distribuídos em dois grupos: AI e OI. Ambos os grupos foram avaliados durante 12 meses pela Escala Visual Analógica (EVA), a Escala de Pontuação do Joelho de Lysholm e pela parte I (pontuação de dor) e a parte II (pontuação de função) da escala Knee Society Score (KSS).

**Resultados** No segundo dia de pós-operatório, a pontuação média na EVA foi significativamente maior no grupo OI ( *p* = 0,0001), mas insignificante ( *p* 0,807) às 6 semanas. Aos 3, 6 e 12 meses de acompanhamento, a pontuação na Escala de Lysholm ( *p* = 0,001) foi significativamente maior no grupo AI. Aos 6 meses, ambas as partes da KSS apresentam uma diferença significativa, com o grupo AI apresentando um desfecho melhor ( *p* = 0,04). No entanto, aos 12 meses, o grupo AI apresentou uma pontuação melhor na parte I da KSS, mas não foram observadas diferenças na parte II.

**Conclusão** Em um acompanhamento de 12 meses, os pacientes submetidos à técnica AI apresentaram melhores pontuações de desfecho e alívio da dor do que aqueles submetidos à técnica OI.

## Introdução

A reconstrução artroscópica do ligamento cruzado anterior (LCA) apresenta vantagens, como um procedimento menos invasivo e uma recuperação mais precoce. Muitos hospitais estão fazendo reconstrução artroscópica do LCA como cirurgia ambulatória. O procedimento pode ser feito pela técnica *all-inside* (AI), que depende apenas da alça ajustável, ou pela técnica *outside-in* (OI), com um túnel tibial completo usando um parafuso de interferência e alça ajustável. 1

Na técnica OI, o enxerto é fixado com um parafuso de interferência no lado tibial e Endobutton (Smith & Nephew, Watford, Hertfordshire, Inglaterra) no lado femoral. Estudos biomecânicos 2 mostraram que os parafusos de interferência têm baixa resistência de fixação devido ao deslizamento do enxerto. Outras preocupações associadas aos parafusos de interferência são a maceração do enxerto e o pequeno contacto entre o tendãoe o osso para a incorporação biológica do enxerto. 2

Na técnica AI, duas concavidades ósseas são feitas em cada lado do enxerto em vez dos túneis ósseos completos. O autoenxerto é suturado de forma simples e contínua em ambos os lados, e as suturas são fixadas ao Endobutton cortical em ambos os lados. 3 Recentemente, entrou em prática o uso de Endobutton cortical com alça ajustável nos lados femoral e tibial, que apresenta a vantagem de um enxerto de tendão sem implante para a fixação óssea nos lados femoral e tibial, o que aumenta a integração biológica entre tendão e osso. 4 Uma causa de preocupação com a técnica AI é o alongamento do enxerto, o que leva a um aumento do espaço entre os dois Endobuttons corticais, o que provoca uma recorrência da frouxidão e instabilidade na articulação do joelho. Além disso, pode levar ao deslizamento do enxerto. 5 Na literatura, há poucos estudos que comparam ambos os métodos em relação aos resultados funcionais; apenas dois ensaios clínicos controlados e randomizados 6 7 e dois estudos prospectivos 8 9 compararam essas duas técnicas. Não há estudos que comparem ambos os métodos utilizando o autoenxerto. Este ensaio clínico controlado e randomizado teve como objetivo comparar os resultados funcionais da reconstrução do LCA pelas técnicas AI e OI.

## Materiais e Métodos

## Desenho do Estudo

Este estudo prospectivo, randomizado e duplo-cego, com dois braços paralelos, foi realizado em um hospital terciário de fevereiro de 2019 a fevereiro de 2022 e aprovado pelo Comitê de Ética (TESE IEC/AIIMS BBSR/PG/2018-19/38).

## Critérios de Inclusão

➢ Pacientes com ruptura do LCA sem melhora após o tratamento conservador.
➢ Pacientes com mais de 90° de flexão do joelho.
➢ Ruptura do LCA associada a lesões de ruptura do menisco medial ou lateral.
➢ Doentes com idades entre 20 e 60 anos.
➢ Rupturas do LCA com histórico de lesão no prazo de um ano.

## Critérios de Exclusão

➢ Pacientes com lesão multiligamentar no joelho.
➢ Pacientes com lesões ósseas associadas dos membros inferiores e lesões da coluna vertebral.
➢ Casos de lesão condral de grau II ou superior, de acordo com a classificação Outerbridge.
➢ Revisão de reconstrução do LCA.
➢ Pacientes com lesões cutâneas no sítio cirúrgico.

## Randomização e Ocultação de Alocação

Os pacientes com LCA que cumpriam com os critérios de inclusão foram recrutados após a assinatura do termo de consentimento livre e esclarecido. Os pacientes foram randomizados em dois grupos por meio de uma programa de randomização on-line ( www.randomization.com ). Sexo, idade, lado lesionado, presença de lesões meniscais ou condrais e duração da lesão foram registrados. Dois cirurgiões (BPP) e (SKP) fizeram as cirurgias de acordo com a alocação. O pesquisador (GD) que avaliou e acompanhou os pacientes nos períodos pré e pós-operatório no ambulatório também foi cegado quanto ao procedimento realizado. Após o procedimento, os pacientes foram avaliado de acordo com o protocolo de pós-operatório. A sequência de randomização foi feita por outro pesquisador (VP), que não esteve envolvido na cirurgia, no recrutamento ou na avaliação pós-operatória dos pacientes.

O tamanho da amostra foi calculado com base na diferença média de 2 com um erro alfa de 5% (intervalo de confiança de 95%). Com base no poder de 80%, verificou-se que o tamanho da amostra era de 19 em cada grupo. Adicionando 15% de abandono a 19, que é 3, O tamanho da amostra obtido foi 22 em cada grupo.

## Técnica Cirúrgica

Em todos os casos, artroscopia diagnóstica de rotina foi feita usando portais padrão. Os autoenxertos de semitendíneo e de grácil foram colhidos para a técnica OI, e somente o enxerto de semitendíneo foi colhido para a técnica AI. Os túneis do osso femoral são feitos de forma semelhante em ambas as técnicas, usando gabaritos padrão. No entanto, o túnel tibial para a técnica AI foi feito com uma broca FlipCutter (Arthrex, Inc., Naples, FL, Estados Unidos) e, para a técnica OI por gabarito padrão. Na técnica AI, o autoenxerto com sutura simples contínua é mantido pelo Endobutton cortical nos lados femoral e tibial, ao passo que, na técnica OI, o autoenxerto é mantido pelo Endobutton cortical no lado femoral e pelo parafuso de interferência no lado tibial. Quaisquer lesões meniscais ou condrais associadas foram desbridadas ou parcialmente ressecadas juntamente com a reconstrução do índice. . Após a cirurgia, foi realizada lavagem do joelho seguida de fechamento da ferida. Uma joelheira estendida foi usada nos ambos os grupos. Após o procedimento, todos os pacientes foram enviados para reabilitação de acordo com o protocolo pós-operatório.

## Reabilitação

Todos os pacientes foram submetidos ao protocolo de reabilitação acelerada, com exercícios de amplitude de movimento (ADM) em cadeia fechada e suporte de peso completo com a joelheira longa a partir do segundo dia de pós-operatório, e exercícios de ADM de cadeia aberta e suporte de peso completo sem joelheira após duas semanas. Depois de duas semanas, eles foram instruídos a fazer reabilitação em casa de acordo com o protocolo. Os pacientes seguiram o protocolo de reabilitação e em geral retornaram ao esporte aos seis meses.

## Medidas de Resultados

Ambos os grupos de pacientes foram avaliados antes da cirurgia e no segundo e quinto dias, na segunda e sexta semanas, e no terceiro, sexto e décimo segundo meses após a cirurgia. As pontuações na Escala Visual Analógica (EVA), na Escala de Pontuação do Joelho de Lysholm e na escala Knee Society Score (KSS) foram avaliadas por um investigador cego (GD) no terceiro, sexto e décimo segundo meses mediante uma pró-forma apropriada. A dor dos pacientes foi classificada com base na pontuação na EVA como leve (< 3), moderada (# a 7) e grave (> 7). A análise de regressão linear foi feita com relação à pontuação na EVA (variável dependente) no segundo dia e a técnica (variável independente).

De acordo com a análise de poder, foram incluídos 44 pacientes (38 homens e 6 mulheres) distribuídos aleatoriamente em 2 grupos (AI e OI) de 22 pacientes ( Fig. 1 ).

**Fig. 1:** Diagrama dos Padrões Consolidados para Relatos de Ensaios (Consolidated Standards of Reporting Trials, CONSORT, em inglês).

## Metodologia Estatística

A análise estatística foi com o programa IBM SPSS Statistics for Windows (IBM Corp., Armonk, NY, Estados Unidos).

## Resultados

## Dados demográficos e Outros Dados de Referência

Não houve diferença significativa entre os dois grupos em termos dos dados demográficos (idade, sexo e índice de massa corporal, IMC) e dos outros dados de referência (duração da lesão, lateralidade, atividade física, lesão meniscal associada) ( Tabela 1a ).

**Tabela 1a:** Dados demográficos de referência

	Grupo AI (n = 22)	Grupo OI (n = 22)
Proporção de sexo masculino:feminino	19:3	19:3
Idade (anos)	29	28,86
Duração da lesão (dias)	150	159
Proporção de lado direito:esquerdo	11:11	12:10
Média do índice de massa corporal (kg/m ^2^ )	24,79	24,79
Atividade física	Sedentarismo: 2; média: 15; praticante de esportes: 5	Sedentarismo: 3; média: 15; praticante de esportes: 4
Lesão meniscal associada	8	7

Abreviaturas: AI, técnica *all-in* ; OI, técnica *outside-in* .

A pontuação média na EVA no segundo dia de pós-operatório foi significativamente mais elevada no grupo OI ( *p* = 0,0001). No entanto, as médias das pontuações na EVA de ambos os grupos na sexta semana de pós-operatório não foram significativamente diferentes ( *p* = 0,807) ( Tabela 1b ).

**Tabela 1b:** Pontuação na EVA de referência e com dois dias e seis semanas de seguimento

Dados	AI (média ± DP)	OI (média ± DP)	IC95% (inferior-superior)		Valor de *p*
EVA de referência	0,45 ± 0,510	0,41 ± 0,503	-0,263	-0,263	0,767
EVA no dia 2	1,32 ± 1,359	4,27 ± 1,579	-3,851	-2,058	0,0001
Diferença média	0,86 ± 1,521	3,86 ± 1,612	-3,963	-2,037	0,0001
EVA em 6 semanas	0,14 ± 0,640	0,18 ± 0,588	-0,419	0,329	0,807
Diferença média	-0,32 ± (0,894)	-0,23 ± 0,869	-0,627	0,445	0,734

Abreviaturas: AI, técnica *all-in* ; DP, desvio padrão; EVA, Escala Visual Analógica; IC95%, intervalo de confiança de 95%; OI, técnica *outside-in* .

## Análise de Regressão Linear

O modelo de análise de regressão linear foi significativo, com um valor R ^2^ de 0,513 ( *p* < 0,05). A mudança da técnica de AI para OI alterou a pontuação na EVA em 2,995 ( Tabela 2 ). Sexo, IMC, idade e atividade física também foram comparados com relação às pontuações na EVA e nas escalas de Lysholm e KSS, mas não resultaram em um modelo significativo.

**Tabela 2:** Análise de Regressão Linear

	R ^2^	B	Valor de *p*
Técnica	0,513	2,955	< 0,05
Pontuação na Escala Visual Analógica			

No terceiro, sexto e décimo segundo meses de seguimento, a pontuação na escala de Lysholm ( *p* = 0,001) foi significativamente maior no grupo AI ( Tabela 3 ). Nessa escala, o desfecho funcional é classificado como ruim (pontuação < 65), regular (pontuação de 65 a 83), bom (pontuação de 84 a 90) e excelente (pontuação > 90). Aos 3 meses, somente 1 paciente no grupo AI teve uma pontuação ruim, em comparação com 5 no grupo OI, e 21 no grupo AI tiveram uma pontuação moderada, em comparação com 17 no grupo OI. Aos 6 meses, 1 paciente do grupo AI teve uma pontuação moderada, em comparação com 4 no grupo OI; 9 pacientes do grupo AI tiveram uma pontuação boa, em comparação com 17 no grupo OI; e 12 pacientes do grupo AI tiveram uma pontuação excelente, em comparação com 1 no grupo OI. Aos 12 meses de seguimento, todos os pacientes, exceto 1 em cada grupo, tiveram pontuações maiores de desfecho funcional, e 1 em cada grupo teve uma pontuação boa. A escala KSS tem duas partes: a pontuação de dor (parte I) e a pontuação funcional (parte II). Houve uma diferença significativa na pontuação na parte I aos 3 meses ( *p* = 0,009), mas não na parte II. Aos 6 meses, observou-se uma diferença substancial entre os dois grupos nas duas partes da escala KSS, e o grupo AI teve pontuação melhor ( *p* = 0,04). No entanto, aos 12 meses de seguimento, o grupo AI teve pontuações significativamente melhores na parte I da escala KSS ( *p* = 0,033); com relação à parte II, não houve diferença significativa entre os grupos ( *p* = 0,543) ( Tabela 4 ). No entanto, ambos os grupos melhoraram significativamente na comparação entre as avaliações pré-operatórias e o seguimento. Os testes da gaveta anterior e de Lachman não mostraram diferença significativa na frouxidão entre os dois grupos, tanto no pré-operatório quanto no seguimento. No entanto, houve uma diferença substancial na frouxidão no pré-operatório e no seguimento em ambos os grupos de acordo com os testes de Lachman e da gaveta anterior.

**Tabela 3:** Pontuação na escala de Lysholm de referência, e com 3, 6 e 12 meses de seguimento

Dados	AI (média ± DP)	OI (média ± DP)	Valor de *p*
Valor de referência	70,73 ± 0,456	70,64 ± 0,492	0,529
3 meses	73,45 ± 4,906	68,14 ± 5,445	0,001
Diferença média após 3 meses	2,73 ± 4,939	-2,50 ± 5,510	0,002
6 meses	90,32 ± 4,202	85,64 ± 4,100	0,001
Diferença média após 6 meses	19,59 ± 4,317	15,00 ± 4,220	0,001
12 meses	97,95 ± 2,7	94,73 ± 3,33	0,001
Diferença média após 12 meses	27,22 ± 0,116	24,09 ± 0,119	0,032

Abreviaturas: AI, técnica *all-in* ; DP, desvio padrão; OI, técnica *outside-in* .

**Tabela 4:** Pontuação na escala Knee Society Score (KSS) de referência, e com 3, 6 e 12 meses de seguimento

Dados	AI (média ± DP)	OI (média ± DP)	Valor de *p*
Parte I da KSS (valor de referência)	70,68 ± 0,477	70,59 ± 0,503	0,512
Parte II da KSS (valor de referência)	69,68 ± ,477	69,50 ± 0,512	0,230
Parte I da KSS (3 meses)	66,73 ± 5,978	60,73 ± 8,430	0,009
Parte II da KSS (3 meses)	51,64 ± 8,963	48,18 ± 9,825	0,230
Diferença média na parte I da KSS (3 meses)	-3,95 ± 5,900	-9,86 ± 8,571	0,011
Diferença média na parte II da KSS (3 meses)	-18,05 ± 8,941	-21,32 ± 9,544	0,247
Parte I da KSS (6 meses)	90,18 ± 2,986	86,73 ± 7,052	0,040
Parte II da KSS (6 meses)	88,23 ± 5,228	84,55 ± 6,710	0,049
Diferença média na parteI da KSS (6 meses)	19,50 ± 2,988	16,14 ± 6,951	0,043
Diferença média na parte II da KSS (6 meses)	18,55 ± 5,180	15,05 ± 6,586	0,050
Parte I da KSS (12 meses)	97,18 ± 3,14	94,14 ± 2,8	0,003
Parte II da KSS (12 meses)	98,45 ± 3,18	97,95 ± 2,5	0,556
Diferença média na parte I da KSS (12 meses)	26,50 ± 1,9	23,55 ± 2,88	0,033
Diferença média na parte II da KSS (12 meses)	28,81 ± 1,9	28,45 ± 2,11	0,543

Abreviaturas: AI, técnica *all-in* ; DP, desvio padrão; OI, técnica *outside-in* .

## Complicações

Nenhum dos pacientes foi submetido a cirurgia de revisão. Seis semanas após a cirurgia, e pacientes no grupo AI (22,72%) e 12 no grupo OI (54%) queixaram-se de sensação alterada no joelho; 2 pacientes no grupo OI desenvolveram sensações de corpo estranho; e 1 paciente no grupo AI desenvolveu sinovite. E derrame articular foi desenvolvido por 2 pacientes no grupo AI e 3 no grupo OI. A sensação de corpo estranho, a sinovite e o derrame articular foram resolvidos em todos os pacientes aos três meses de acompanhamento.

## Discussão

Na literatura, há poucos estudos que comparam ambos os métodos em relação aos resultados funcionais; apenas dois ensaios clínicos controlados e randomizados 6 7 e dois estudos prospectivos 8 9 compararam essas duas técnicas. Volpi et al. 9 relataram que, no que diz respeito à função articular adequada e ao retorno ao esporte, não há diferenças entre a técnica AI e a reconstrução tradicional do LCA utilizando tendões do semitendíneo e do grácil. Lubowitz et al. 6 não relataram diferenças nas pontuações no Formulário de Exame do Joelho do International Knee Documentation Committee (IKDC), na escala KSS, e no 12-Item Short-Form Health Survey (SF-12), nem com relação ao alargamento tibial e femoral, mas relataram que a reconstrução pela técnica AI resultou em pontuações menores de dor na EVA mais baixo em comparação com os valores de referência. Em outro estudo, Lubowitz et al. 7 concluíram que não houve diferenças significativas na estabilidade anteroposterior do joelho nem em outros resultados na comparação entre a reconstrução do LCA com aloenxerto por AI usando fixação de abertura e usando fixação suspensa. O comprimento do enxerto demandado pelo grupo AI variou de 6 cm a 6,5 cm, e, no grupo OI, de 8,5 cm a 9cm. A demanda de comprimento do enxerto foi menor no grupo AI, de modo que maior espessura do enxerto fica disponível para a reconstrução ligamentar.

Em uma revisão sistemática publicada em 2018, de Sa et al. 10 relataram resultado funcional melhor e taxa de falha do enxerto baixa com a técnica AI. Browning et al. 11 revisaram reconstrução do LCA usando fixação de abertura ou fixação suspensiva, e observaram que a fixação suspensiva resultou em menos casos de falha do enxerto e melhor estabilidade do joelho. No entanto, no presente estudo, com os testes de Lachman e da gaveta anterior, não observamos diferença significativa na estabilidade do joelho entre os dois grupos, e nenhum de nossos pacientes foi submetido a cirurgia de revisão. A diferença no desfecho funcional entre os dois métodos não foi significativa em estudos anteriores. 6 No entanto, neste estudo, com um seguimento médio de 12 meses, foram observados desfechos funcionais melhores no grupo AI. Descobrimos que a técnica AI propicia um alívio significativo da dor do período pós-operatório imediato até seis semanas. Depois disso, não houve diferença significativa nas pontuações na EVA entre os dois grupos. Benea et al. 12 concluíram que o nível de dor foi menor no grupo AI do que no grupo de fixação cortical clássica em um mês de seguimento. Em 2015, Lubowitz et al. 7 compararam a fixação suspensa usando botões corticais femorais e tibiais e a fixação de abertura usando um parafuso canulado de interferência femoral e um parafuso canulado de interferência retrógrada tibial; o seguimento foi de dois anos, e a “medida de desfecho primária foi estabilidade anteroposterior (AP) do joelho medida por meio do dispositivo KT-1000 (MEDmetric, San Diego, CA). As medidas de desfecho secundárias incluíram mudanças na pontuação de dor na Escala Visual Analógica *versus* o período pós-operatório, o consumo de narcóticos, a classificação de exame do joelho do International Knee Documentation Committee, a pontuação de avaliação subjetiva do International Knee Documentation Committee, as pontuações na escala Knee Society Score e no Short-Form 12, e análise radiográfica para o alargamento da concavidade”. Os autores 7 não encontraram diferenças significativas em nenhuma das escalas de desfecho. Em 2014, Volpi et al. 9 compararam a dor e o desfecho funcional usando as escalas de Tegner e de Lysholm e a pontuação do IDKC com relação à reconstrução feita pela técnica transtibial AI e pela técnica transtibial tradicional, com acompanhamento de dois anos, e não observaram mudanças significativa em nenhum dos desfechos. O retorno ao esporte é uma importante medida de desfecho após a reconstrução do LCA; no entanto, depende do tipo de esporte e do protocolo de reabilitação. As taxas de falha do enxerto relatadas para a reconstrução do LCA pela técnica AI na literatura 3 14 15 16 variam de 4,9% a 12,7%; os mecanismos que levaram à falha nesses estudos foram traumas ou lesões relacionadas com o esporte.

Para tirar conclusões sobre a taxa de falha do enxerto, o presente estudo precisa de mais tempo de seguimento. No entanto, Connaughton et al. 3 relataram resultados globais semelhantes em estudos de desfechos subjetivos e objetivos, mas uma taxa elevada de falha do enxerto com a técnica AI. No entanto, seu estudo 3 contém viés, pois eles coletaram apenas aloenxertos para a reconstrução, que têm uma alta propensão a falhar em indivíduos jovens ativos. Pallis et al. 17 observaram taxas elevadas de revisão em reconstruções do LCA usando aloenxerto, e recomendaram autoenxerto para a reconstrução do LCA em atletas jovens. Em 2017, Schilaty et al., 18 avaliaram a incidência de segunda lesão do LCA e os fatores de risco associados a ela, e descobriram que os aloenxertos estão associados a um risco maior de falha do enxerto quando comparados aos autoenxertos isquiotibiais e de tendão patelar-osso. Não há dados conclusivos na literatura sobre as taxas de falha do enxerto na comparação entre as técnicas AI e OI com autoenxerto. No entanto, a probabilidade de falha do enxerto é maior com o autoenxerto dos isquiotibiais se o diâmetro do enxerto for inferior a 8 mm. 19 A técnica OI utiliza autoenxerto do semitendíneo e do grácil, ao passo que o tendão do grácil é poupado na técnica AI. Magnuseen et al. 19 concluíram que a coleta do tendão do grácil afeta negativamente o torque isocinético de flexão do joelho a uma baixa velocidade angular. Esse achado é significativo para esportes que requerem alta atividade funcional de modo que os autores 19 recomendaram a preservação do tendão do grácil sempre que possível. A técnica AI também resulta em morbilidade menor no local do enxerto, uma vez que o tendão do grácil é poupado. 20 Kouloumentas et al. 21 descreveram uma melhor preservação da força de flexão do joelho na reconstrução do LCA pela AI do que pela técnica OI convencional. Monaco et al. 22 afirmaram que a técnica poupadora do grácil é minimamente invasiva para reconstrução do LCA e resulta em uma melhor força de flexão em baixa velocidade angular do que a técnica do túnel tibial completo.

Todos os pacientes de ambos os grupos haviam sido submetidos a uma reabilitação acelerada. 23

As limitações deste estudo são o tamanho relativamente pequeno da amostra e o seguimento de apenas um ano, o que pode explicar a ausência de casos de falha do enxerto nos nossos pacientes.

Nossos achados mostram que o grupo AI apresentou menos dor e melhores desfechos funcionais quando seguido por 12 meses em comparação com o grupo OI, e que ambas as técnicas são bem-sucedidas na restauração da estabilidade ligamentar do joelho, e resultam em boas medidas de desfechos relatados pelos pacientes e alívio da dor em comparação aos níveis pré-operatórios.

## Resultado Principal

O grupo AI apresentou desfechos funcionais melhores do que os do grupo OI.

## Conclusão

Neste ensaio clínico controlado e randomizado, descobrimos que as técnicas AI e OI com autoenxerto de isquiotibiais para reconstrução do LCA resultaram na restauração da estabilidade do joelho e em boas medidas de desfecho relatadas pelos pacientes. No entanto, o grupo AI teve pontuações melhores na escala de Lysholm e mais alívio da dor em um seguimento de 12 meses.
